# Evidence of survival bias in the association between *APOE-Є4* and age at ischemic stroke onset

**DOI:** 10.3389/fgene.2024.1392061

**Published:** 2024-09-02

**Authors:** Joanna von Berg, Patrick F. McArdle, Paavo Häppölä, Jeffrey Haessler, Charles Kooperberg, Robin Lemmens, Alessandro Pezzini, Vincent Thijs, Sara L. Pulit, Steven J. Kittner, Braxton D. Mitchell, Jeroen de Ridder, Sander W. van der Laan

**Affiliations:** ^1^ Center for Molecular Medicine, Division Laboratories, Pharmacy, and Biomedical Genetics, University Medical Center Utrecht, Utrecht University, Utrecht, Netherlands; ^2^ Oncode Institute, Utrecht, Netherlands; ^3^ Division of Endocrinology, Diabetes and Nutrition, Department of Medicine, University of Maryland School of Medicine, Baltimore, MD, United States; ^4^ Institute for Molecular Medicine Finland FIMM, HiLIFE, University of Helsinki, Helsinki, Finland; ^5^ Division of Public Health Sciences, Fred Hutchinson Cancer Center, Seattle, WA, United States; ^6^ University Hospitals Leuven, Department of Neurology, Leuven, Belgium; ^7^ KU Leuven–University of Leuven, Department of Neurosciences, Experimental Neurology, Leuven, Belgium; ^8^ Department of Medicine and Surgery, University of Parma, Parma, Italy; ^9^ Stroke Care Program, Department of Emergency, Parma University Hospital, Parma, Italy; ^10^ Stroke Theme, The Florey, Heidelberg, VIC, Australia; ^11^ Department of Medicine, University of Melbourne, Melbourne, VIC, Australia; ^12^ Department of Neurology, Austin Health, Heidelberg, VIC, Australia; ^13^ Geriatric Research and Education Clinical Center, VA Maryland Healthcare System, Baltimore, MD, United States; ^14^ Department of Neurology, University of Maryland School of Medicine, Baltimore, MD, United States; ^15^ Central Diagnostics Laboratory, Division Laboratories, Pharmacy, and Biomedical Genetics, University Medical Center Utrecht, Utrecht University, Utrecht, Netherlands; ^16^ Center of Population Health and Genomics, University of Virginia, Charlottesville, VA, United States

**Keywords:** genome-wide association study, stroke, age at onset, *APOE*, atherosclerosis

## Abstract

**Introduction:**

Large genome-wide association studies (GWASs) using case–control study designs have now identified tens of loci associated with ischemic stroke (IS). As a complement to these studies, we performed GWAS in a case-only design to identify loci influencing the age at onset (AAO) of ischemic stroke.

**Methods:**

Analyses were conducted in a discovery cohort of 10,857 ischemic stroke cases using a linear regression framework. We meta-analyzed all SNPs with *p*-value <1 x 10^−5^ in a sexcombined or sex-stratified analysis using summary data from two additional replication cohorts.

**Results:**

In the women-only meta-analysis, we detected significant evidence for the association of AAO with rs429358, an exonic variant in apolipoprotein E (*APOE*) that encodes for the APOE-Є4 allele. Each copy of the rs429358:T>C allele was associated with a 1.29-year earlier stroke AAO (meta *p*-value = 2.48 x 10^−11^). This APOE variant has previously been associated with increased mortality and ischemic stroke AAO. We hypothesized that the association with AAO may reflect a survival bias attributable to an age-related decrease in mortality among APOE-Є4 carriers and have no association to stroke AAO per se. A simulation study showed that a variant associated with overall mortality might indeed be detected with an AAO analysis. A variant with a 2-fold increase in mortality risk would lead to an observed effect of AAO that is comparable to what we found.

**Discussion:**

In conclusion, we detected a robust association of the APOE locus with stroke AAO and provided simulations to suggest that this association may be unrelated to ischemic stroke per se but related to a general survival bias.

## Introduction

Genetic association analysis and, in particular, genome-wide genetic association studies (GWASs) have become standard tools for identifying genetic contributions to complex diseases, including ischemic stroke (IS). Although such studies are typically framed as case–control study designs, case-only designs have also been used, for example, for the purpose of identifying variants that are associated with the age at disease onset, i.e., timing of disease. Such associations may reflect variants that predispose to earlier forms of disease or modify the effects of other disease-predisposing variants. Case-only age at onset association analyses have been carried out on multiple traits, including Alzheimer’s disease (Naj et al., 2014), Parkinson’s disease (Blauwendraat et al., 2019), and stroke (Lagging et al., 2019). Case–control approaches that condition on clinical covariates, such as the age at onset (AAO), have also been used to boost the power to detect risk loci for complex diseases (Zaitlen et al., 2012; Traylor et al., 2014).

To identify variants associated with ischemic stroke AAO, we performed a two-stage meta-analysis of GWAS for AAO in 10,857 stroke cases from the Stroke Genetics Network (SiGN) (Meschia et al., 2013), followed by a replication of all associated SNPs (*p*-value <5 × 10^−5^) in two independent studies, FinnGen Data Freeze 5^7^ and the Women’s Health Initiative (WHI) (The WHI Study Group, 1998). We performed sex-combined and sex-stratified analyses. From these analyses, we identified a variant in the apolipoprotein E (*APOE*) locus, encoding the ApoE-Є4 allele, that was significantly associated with earlier AAO in women (rs429358:T>C, meta *p*-value = 2.48 × 10^−11^, beta = 1.29 ± 0.38 years) but not in men. This SNP has previously been associated with stroke AAO in a prior candidate gene study (Lagging et al., 2019), yet, it is not associated with the risk of ischemic stroke (OR = 1.0, 95% confidence interval [CI], 0.97–1.03, and *p* = 0.97; n_cases_ = 33,936 vs n_controls_ = 391,114 in European populations (Malik et al., 2018)). Thus, we hypothesized that the association with earlier stroke AAO may reflect an overall association of this variant with earlier death (Pilling et al., 2017). To test this hypothesis, we performed a simulation study in which we simulated loci that are associated with overall mortality unrelated to stroke to characterize parameters that would lead to age-related differences in allele frequencies that could be misinterpreted as being related to the age at disease onset.

## Results

A summary of the characteristics of the discovery (SiGN) and follow-up replication cohorts (FinnGen Data Freeze 5 and WHI) is shown in Table 1. The mean age of stroke onset was 67.6, 66.7, and 76.6 years, in SiGN, FinnGen, and WHI (the latter includes only women), respectively.

**TABLE 1:** Characteristics of the study populations. Percentages have been calculated on the subset of individuals with no missing values for the variable in question. Hypertensio*n*: as derived from multiple administrative registers. CAD, coronary artery disease. T2*D*, type 2 diabetes. DF*5*, FinnGen Data Freeze 5^7^. Stratification was based on self-reported sex.

Study	Variable	Sex-combined	Men	Women
SiGN	Sample size	12,145	6,801	5,344
	Age at onset (mean ± sd) in years	67.6 ± 14.6	65.5 ± 13.8	70.2 ± 15.3
	Hypertension	7,888 (65.0%)	4,269 (62.8%)	3,619 (67.8%)
	CAD	2,401 (19.8%)	1,438 (21.2%)	963 (18.0%)
	T2D	2,757 (22.7%)	1,598 (23.5%)	1,159 (21.7%)
	Current smoker	2,665 (22.0%)	1,715 (25.3%)	950 (17.8%)
FinnGen DF5	Sample size	8,124	4,708	3,416
	Age at onset (mean ± sd) in years	66.7 ± 13.3	66.0 ± 12.5	67.8 ± 14.3
	Hypertension	5,274 (64.9%)	3,017 (64.1%)	2,257 (66.1%)
	CAD	2,582 (31.8%)	1,775 (37.7%)	807 (23.6%)
	T2D	2,648 (32.6%)	1,645 (34.9%)	1,003 (29.4%)
	Current smoker	-	-	-
WHI	Sample size	-	-	3,415
	Age at onset (mean ± sd) in years	-	-	76.6 ± 7.1
	Hypertension	-	-	2,720 (79.65%)
	CHD	-	-	821 (24.04%)
	T2D	-	-	846 (24.77%)
	Current smoker	-	-	258 (7.55%)

**FIGURE 1:**
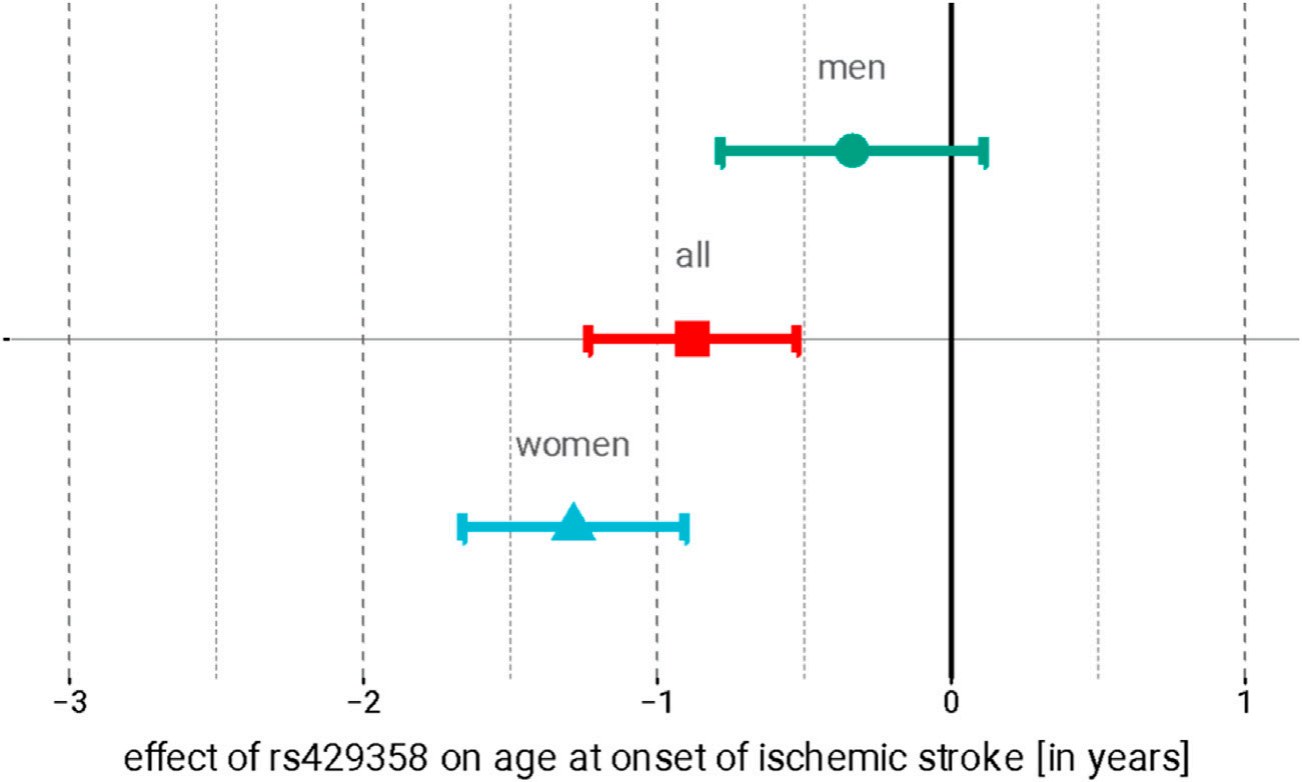
Association of APOE rs429358 with the age at stroke onset in women (red), men (blue), and sex-combined meta-analyses. Point estimate represents the effect of each copy of the minor (C) allele on the AAO (in years); 95% confidence intervals are indicated by error bars.

In the SiGN discovery cohorts, genome-wide association analyses revealed 61 individual loci associated with stroke AAO in the sex-combined analysis at a significance threshold of *p* <1.0 × 10^−5^ (see Methods), 144 individual loci in the men-only analysis, and 37 individual loci in the women-only analysis (Supplementary Figure S1; Supplementary Figure S2). There was one genome-wide significant hit (rs6051656) in the men-only analyses in SiGN spanning a 14-kb region at chromosome 20:364,295–378,978 near TRIB3 (Supplementary Figure S3; Supplementary Table S1), which did not replicate in the men-only meta-analysis of SiGN with FinnGen (Supplementary Table S1). Further meta-analyses of all SNPs associated at *p* <1.0 × 10^−5^ in SiGN with FinnGen did not yield genome-wide significant results for the sex-combined analysis but did reveal a genome-wide significant association in the women-only analysis with rs429358 on chromosome 19 at the *APOE* locus (p_meta,SiGN-FinnGen_-value = 2.4 × 10^−8^, beta = −1.63 years ±0.29; Table 2; Supplementary Figure S4). This SNP was further replicated in the WHI (p_meta,SiGN-FinnGen-WHI_ = 2.48 × 10^−11^, beta = −1.29 ± 0.38). APOE-rs429358 is associated with stroke AAO in both sexes combined, although the magnitude of association is stronger in women than in men (unequal variance *t*-test, *p* = 4.3 × 10^−4^; Figure 1). Conditional analysis (using the SiGN summary statistics) indicated no secondary associated SNPs at this locus.

**TABLE 2:** Meta-analysis results, total sample and sex-specific, of the association of *APOE*-rs429358 (chr19:45,411,941, b37) with the ischemic stroke age at onset in discovery (SiGN) and replication (FinnGen and WHI) cohorts**.** The sample size is for the total sample and for men/women. The *alleles* are the effect (C) allele and other (T) allele with the corresponding effect allele frequency (EAF). The beta (effect size) and *se* (standard error) correspond to the difference in the age at onset (in years) associated with each copy of the risk allele (C). As the WHI includes only women, the results are presented for “women-only.”

Group	Discovery				Replication								Meta-analysis			
	SiGN (n = 10,857; 6,178/4,679)				FinnGen (n = 8,124; 4,708/3,416)				WHI (n = 3,415; 0/3,415)				(n = 22,126; 10,886/11,510)			
	EAF	Beta	se	*p*-value	EAF	Beta	se	*p*-value	EAF	Beta	se	*p*-value	EAF	Beta	se	*p*-value
Sex-combined	0.13	−1.21	0.25	1.60 × 10^−6^	0.18	−0.53	0.26	0.04	-	-	-	-	0.16	−0.88	0.18	1.19 × 10^−6^
Men-only	0.14	−0.80	0.32	0.01	0.18	0.13	0.32	0.68	-	-	-	-	0.16	−0.34	0.23	0.14
Women-only	0.13	−1.78	0.40	9.70 × 10^−6^	0.17	−1.47	0.43	5.70 × 10^−4^	0.13	−1.02	0.26	6.97 × 10^−5^	0.14	−1.29	0.19	2.48 × 10^−11^

*APOE* rs429358 is a missense variant for which the minor allele, C, changes the amino acid at the 112th position of the ApoE protein from a cysteine to an arginine, thus altering the protein conformation. In combination with a second SNP (rs7412:C>T) in *APOE*, rs429358 encodes the ApoE isoform of an individual, with the rs429358-C allele associated with the ApoE-Є4 allele. The ApoE-Є4 allele (frequency of 0.16 in SiGN) has been associated with numerous adverse health outcomes, including hyperlipidemia (Khalil et al., 2021) and lipid metabolism (Graham et al., 2021), Alzheimer’s disease (Serrano-Pozo et al., 2021) and dementia, and coronary disease (Aragam et al., 2022). However, this variant is neither associated with ischemic stroke susceptibility in MEGASTROKE (OR = 1.00; 95% CI: 0.96–1.03; *p* = 0.77) nor with any other ischemic stroke subtype (Supplementary Table S2) (Malik et al., 2018).

An association of *APOE* rs429358 with stroke AAO has been reported previously (Deelen et al., 2011), and this variant has also been associated with longevity (Pilling et al., 2017) and the age of parental death (Lew, 1985). These observations, coupled with the association we observed between *APOE* rs429358 and stroke AAO, albeit genome-wide significant in women only, prompted us to investigate via simulation whether the stroke AAO association could be a manifestation of a survival bias attributable to a higher overall mortality among ApoE-Є4 carriers.

**FIGURE 2:**
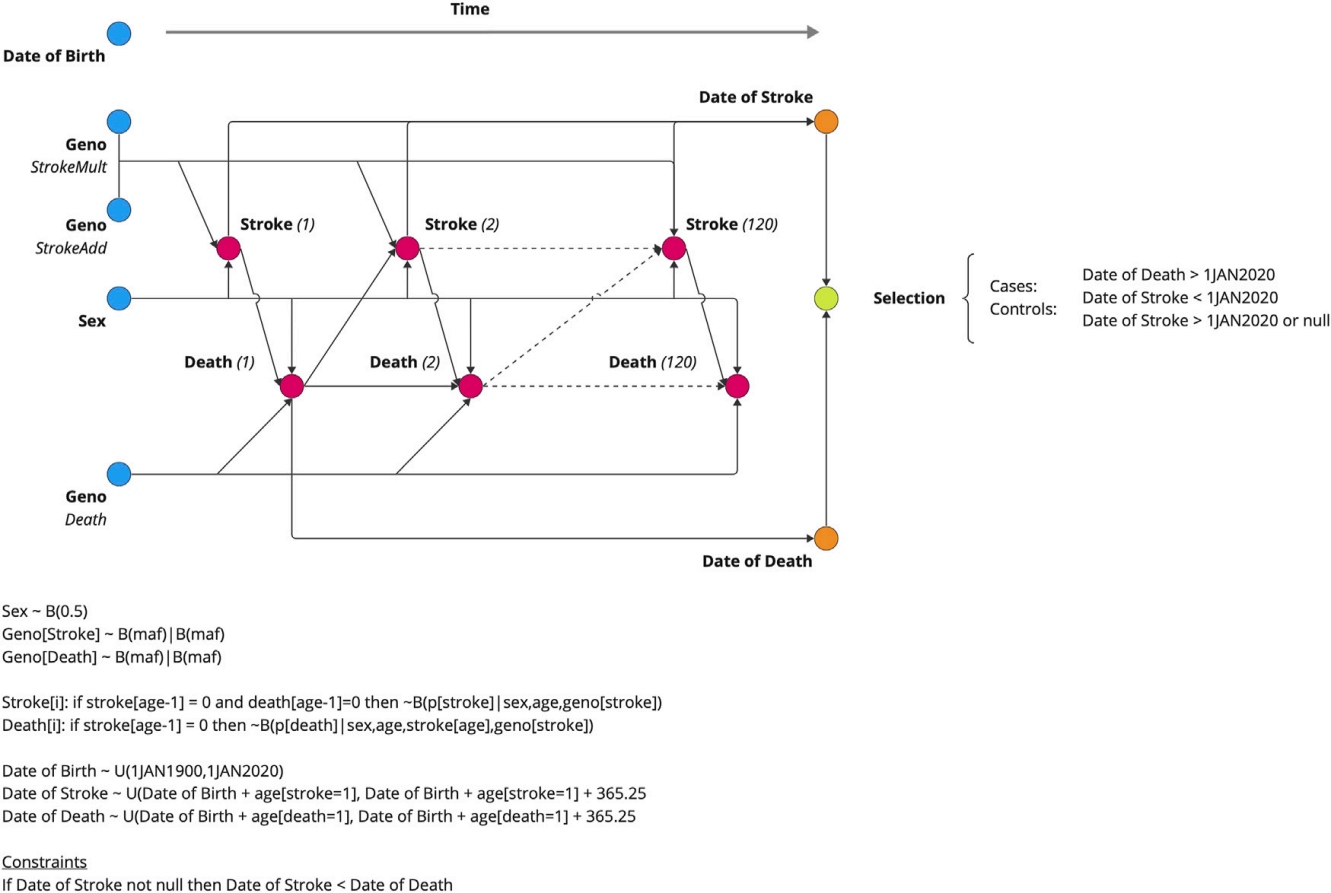
Data generating model for the simulation study. Five variables were generated at birth (DOB, GENO, and SEX), and the subsequent risks of stroke and death were estimated annually. The date of death and date of stroke (if applicable) are outputted from the model. The genotypic effect varied from 1.0 (null) to 2.0 by increments of 0.1. The probability draw parameters for the Markov chain Monte Carlo simulations are given with B(p|c) notation, indicating a draw from the binomial distribution **(B)** with a probability (p) of stroke, given the conditions **(C)**, i.e., sex, age, and geno(types).

We simulated a population of individuals who were followed from birth until death based on age-specific mortality rates obtained from the Social Security Administration Actuarial Life Tables (Figure 2) (Lew, 1985). Birthdates for the simulated subjects were randomly drawn between 1 January 1900 and 1 January 2020. Each individual was assigned a genotype for three SNPs, G_ISmultiplicative_, G_ISadditive_, and G_death_. G_ISmultiplicative_ and G_ISadditive_ increased the risk of ischemic stroke only, and G_death_ increased the risk of death only but not through IS. Stroke was assumed to increase the risk of death as a function of the time since the event. We performed association analyses for each simulated SNP and two phenotypes: logistic regression of case–control status and linear regression of the age at onset. Methods provides the simulation details.

**FIGURE 3:**
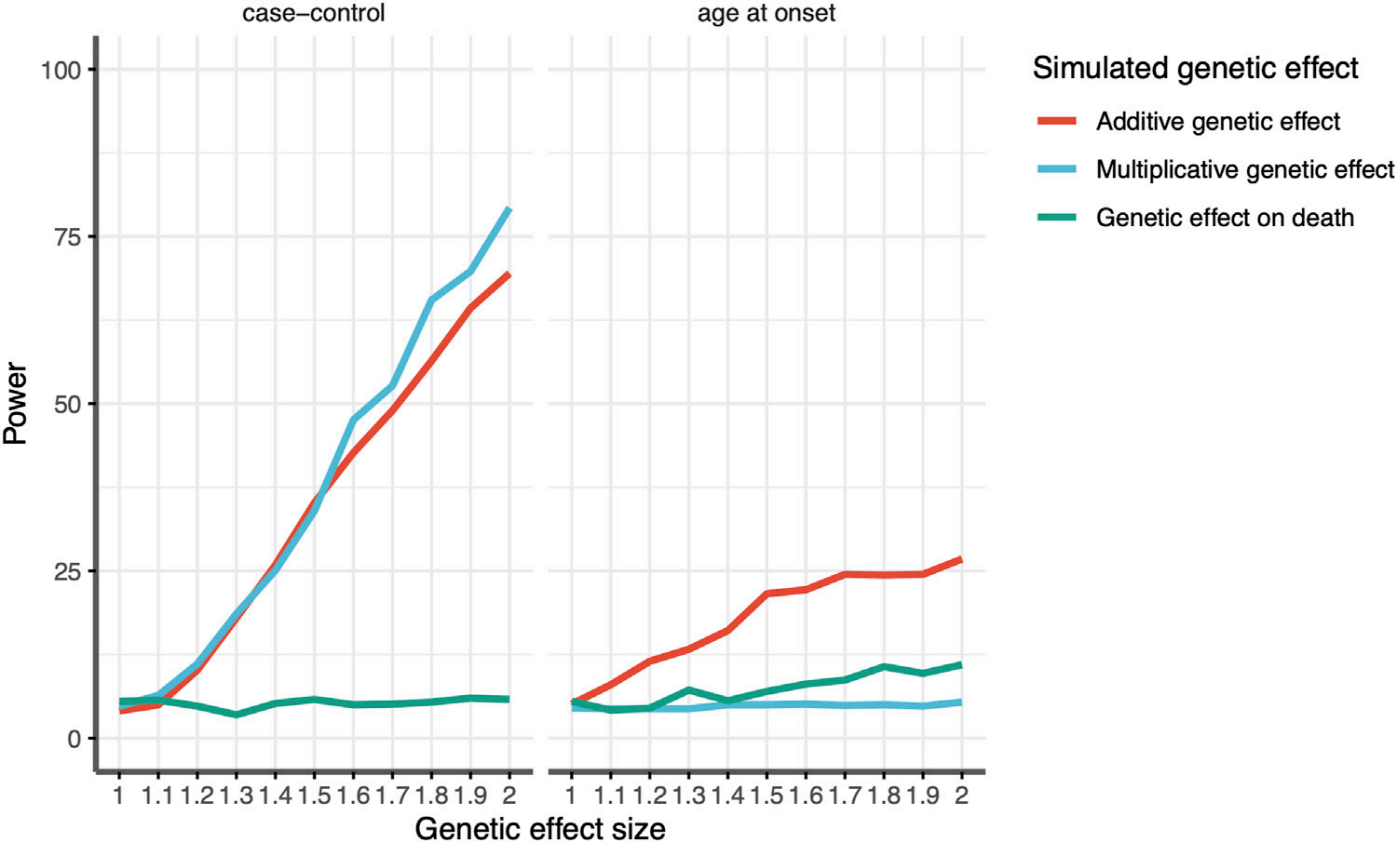
Estimated power to detect an association between the genotype and stroke according to the effect size for variants associated with age at death (green), additive effect on stroke susceptibility (red), and multiplicative effect on stroke susceptibility (blue) for (left) case–control analysis and (right) age-at-onset analysis. The x-axis shows the effect size in the relative risk for a given genetic variant. The y-axis shows the power, given a genetic effect size.

As expected, both the additive and multiplicative loci simulated to influence the risk of stroke were identifiable via a case–control design (1,000 cases and 1,000 controls), with power increasing with larger effect sizes (Figure 3A). Whether they were associated with AAO depended on the amount of risk conferred by the allele (Figure 3B). Loci with a relative increase in risk were not associated with AAO at all, but those with an additive increase in risk saw proportionally more strokes at early ages, and thus, the risk allele was associated with a lower AAO of IS. The simulated locus that was associated with mortality via mechanisms unrelated to stroke was not associated with stroke risk. However, that locus was associated with the AAO. A locus with a 2-fold increase in mortality would display an association with a ∼1.5-year decrease in the age at onset, an effect size similar to that identified for the *APOE* locus in our GWAS. In other words, our results indicate that the observed SNP association in women is biased by an association with earlier death, assuming that the SNP effect is indeed independent of ischemic stroke.

## Discussion

We found that *APOE*-rs429358, encoding the ApoE-Є4 haplotype, is associated with an earlier age at ischemic stroke in women. Although this SNP has not been associated with an increased risk of ischemic stroke in previous GWASs, there are several lines of evidence supporting that the association we observed with stroke AAO is real. First, this association is statistically robust and replicated in two independent populations. Second, this SNP has been associated previously in a candidate gene study with stroke AAO (Gerdes et al., 2000; Sebastiani et al., 2012; Brooks-Wilson, 2013). Third, this SNP is biologically compelling as it encodes the ApoE-Є4 allele, which has been associated previously with a shorter lifespan (Mahley, 2016) and several common age-related conditions, including Alzheimer’s disease (Wilson et al., 1996; Bennet et al., 2007; Mahley, 2016; Li et al., 2021) and ischemic heart disease (Garatachea et al., 2014; Caruso et al., 2022).

Although the association between rs429358 and ischemic stroke AAO appears to be robust, the interpretation of this association can be debated. One possibility is that this variant, while not associated with stroke susceptibility *per se*, is associated with the age at which stroke occurs in individuals more likely (either through genetic or environmental factors) to experience a stroke. A second possibility is that the association of this variant with stroke AAO is a more general consequence of the *APOE* risk allele at this locus being associated with a shortened lifespan in general, i.e., an age-related decrease in the frequency of the risk allele. Our simulation study supports the latter interpretation. In other words, the association we detected between *APOE* and stroke AAO may be due to a survival bias. Our simulations suggest that a locus exerting a two-fold increase in mortality would also be associated with a ∼1.5-year decrease in the age at onset, an effect size similar to that identified for the *APOE* locus in our GWAS and is consistent with previous findings (Martin et al., 2021).

Although previous simulation studies have assessed the power of AAO studies (Gamache et al., 2020), these previous studies have failed to account for the competing risk of mortality and potential bias that may play a role in these studies. Our simulations utilize a prospective data generating model, even though a retrospective case–control or case-only analytic model is fit to the data. This allows our simulations to estimate associations (either OR from a case–control study or year difference in AAO studies) that are more likely to represent those found in the natural population.

The reason for the predominantly larger effect on stroke AAO we observed among females compared to male stroke cases is not apparent. Possible explanations include a disproportionate effect of ApoE-Є4 on survival in women compared to men, a direct effect of ApoE-Є4 on stroke AAO, or chance. Modest differences in the associations of the *APOE* genotype have been reported between men and women for ischemic heart disease (Joshi et al., 2016), Alzheimer’s disease (Corbo and Scacchi, 1999), and lifespan (Finch and Morgan, 2007; Yassine and Finch, 2020), although the reasons underlying these differences are not known. An alternative hypothesis may be more plausible and seems supported by our simulations and GWAS results. It is thought that ApoE-Є4 is the proinflammatory ancestral allele in humans and adaptive to reducing mortality under conditions of infections, food scarcity, and (paradoxically) a shortened life expectancy (NINDS Stroke Genetics Network et al., 2016; Caruso et al., 2022). Several lines of evidence support this notion. ApoE-Є4 shows less severe liver damage during hepatitis C infections, the allele frequency remained high in populations where food was scarce until recently, and the allele is associated with higher circulating cholesterol levels (Caruso et al., 2022). As the human population expanded and thrived, the ApoE-Є3 and ApoE-Є2 alleles spread, while the ApoE-Є4 allele was maintained by balancing selection (NINDS Stroke Genetics Network et al., 2016; Caruso et al., 2022). Yet, as conditions for our species continued to improve, diets and lifespan changed, thus rendering ApoE-Є4 susceptible to cardiovascular disease (CVD) and more pronounced effects in women (Caruso et al., 2022).

Although there is no doubt that the *APOE* locus is an important determinant of atherosclerosis, the largest GWAS, to date, of stroke and its subtypes has revealed no evidence of an association of this locus with stroke risk (Malik et al., 2018). Moreover, our simulation analyses suggest that a plausible explanation of the association of this locus with stroke AAO can be entirely explained by survival bias, an explanation that is entirely consistent with the robust association reported by multiple studies of the APOE locus with lifespan.

Strengths of our study include the large number of well-characterized ischemic stroke cases with a wide range of ages at onset, as well as the prospective design of our simulation study. Nevertheless, our study is not without limitations. One notable result is that the effect size in WHI women (−1.02) is in the same direction but smaller in magnitude, possibly reflecting the fact these women, on average, experienced stroke onset at substantially older ages with a higher prevalence of hypertension and lower current smoking than the other female participants and the men. True as this may be, if anything, the overall meta-analysis effect estimate is smaller, which does not preclude the main interpretation that results are consistent despite variations across studies. However, foremost among the limitations, none of the studies included in our meta-analysis were birth cohorts, so only individuals who survived until the age of their recruitment are part of our GWAS. If case-fatality rates differed between early- and late-onset strokes, then variants specifically associated with early (or late)-onset stroke could go undetected. Likewise, our study may be affected by an ascertainment bias as cases were drawn from case–control studies, i.e., people were ascertained on a phenotype that may introduce confounding effects, such as some strokes that are more likely to be detected by clinicians or the severity of stroke. However, the replication in FinnGen sidesteps this issue because people are randomly ascertained in this study.

In conclusion, we detected a robust association of the *APOE* locus with stroke AAO and provided simulations to suggest that this association may be unrelated to ischemic stroke *per se* but related to a general survival bias.

## Methods

### Genome-wide analysis of stroke age at onset

#### Study populations

Analyses were performed on subjects of the European ancestry from SiGN, FinnGen study, and WHI. SiGN is an international collaboration that includes ischemic stroke cases recruited from multiple sites in the United States and Europe (United Kingdom, Poland, Belgium, Spain, Austria, and Sweden) (Meschia et al., 2013). The discovery phase of this analysis included 10,857 SiGN participants, while the replication phase included 8,124 participants from FinnGen Data Freeze 5^32^ and 3,415 participants from the WHI (Wassertheil-Smoller et al., 2003). Participants provided (written) informed consent, and all studies upheld the ethical standards according to the Declaration of Helsinki.

#### Phenotype definition

For each study included in the SiGN dataset, ischemic stroke was confirmed by neuroimaging. Details, including inclusion criteria, for each study are given in the original SiGN GWAS publication (Adams et al., 1993). In the WHI, all incident strokes, other vascular events, and deaths were identified through a self-report at annual (OS) and semi-annual (CT) participant contacts and through third-party reports by family members and proxies. Medical records were obtained for potential strokes, and adjudication was performed by trained physician adjudicators who assigned a diagnosis. Stroke diagnosis requiring and/or occurring during hospitalization was based on the rapid onset of a neurological deficit attributable to an obstruction or rupture of an arterial vessel system. The deficit was not known to be secondary to brain trauma, tumor, infection, or other causes and must have lasted more than 24 h, unless death supervened or a lesion compatible with acute stroke was evident on computed tomography or a magnetic resonance imaging scan (NINDS Stroke Genetics Network et al., 2016). Strokes were classified as ischemic, hemorrhagic, or unknown/missing. Ischemic stroke subtypes were further classified using Trial of Org 10172 in Acute Stroke Treatment (TOAST) criteria (Regier et al., 2018).

#### DNA isolation, genotyping, and imputation

The SiGN and WHI participants were genotyped on different Illumina platforms. The dataset was divided into different study strata based on similar genetic ancestry and genotyping platforms. Genotypes that were not measured were imputed against 1000G phase 3 for SiGN (Chang et al., 2015) and TOPMed for WHI (1000 Genomes Project Consortium et al., 2015). The FinnGen cohort and methods are comprehensively described elsewhere (Kurki et al., 2023).

#### Genome-wide linear regression

Genome-wide analyses of stroke AAO were carried out in SiGN using BOLT-LMM (1000 Genomes Project Consortium et al., 2015). We used PLINK 1.9^38^ to “hard call” and subset the imputed SNPs to build the genetic relationship matrix (GRM). SNPs with an INFO <0.8, genotyping rate <95%, or missing genotype rate >5% were excluded from the analysis. Covariates in the association analysis included the study stratum, the GRM to correct for subtle population stratification, and sex (in the sex-combined analysis). After the analysis, we filtered out SNPs with minimum minor allele frequency (MAF) <5%.

#### Independent signals

To identify independent signals for the three main analyses in the discovery, we used PLINK 1.9 with 1000G phase 1 (version 3) as a reference (1000 Genomes Project Consortium et al., 2015). We set the minimal *p*-value threshold at 0.05 and defined a clumped region as ±500 kb, with a minimum linkage disequilibrium r^2^ at 0.05 (--clump-p1 5e-8 --clump-p2 0.05 --clump-kb 500 --clump-r2 0.05 --clump-best --clump-verbose). To identify independent signals to take forward in the replication phase, we included clumps at *p* < 1.0 × 10^−5^ (--clump-p1 1e-5 --clump-p2 0.05 --clump-kb 500 --clump-r2 0.05 --clump-best --clump-verbose).

#### Conditional analysis

We used GCTA-COJO (Yang et al., 2012) to investigate whether there were additional associated SNPs at the discovered loci. We used the stepwise model selection procedure (--cojo-slct) and used the imputed genotype data (converted to :hard call,” as described for the GWAS) as input (--bfile).

#### Meta-analysis

We performed a look-up of SNPs that were associated at *p* < 1.0 × 10^−5^ in any of the three analyses (sex-combined, men-only, and women-only) in FinnGen and the Women’s Health Initiative (women-only and for rs429358 only) (Winkler et al., 2015). Meta-analyses of SiGN with FinnGen were performed in METAL using the inverse-variance weighted approach (Lew, 1985). A *p*-value threshold of *p* < 5 × 10^−8^ was considered to be significant in the meta-analysis. Baseline characteristics for the replication datasets are given in Table 1.

#### Testing for sex differences in effect size

To test how likely the differences in effect size between the women and men analyses are, under the null hypothesis of no difference, we used a *t*-test for unequal variance. The test we used is similar to Welch’s *t*-test, but we additionally corrected for Spearman’s rank correlation *r* between all women and men effect sizes (filtered on MAF >0.05 and INFO >0.8)^40^; *r* was equal to 0.018.

$$t=\frac{{beta}_{XX}-\;{beta}_{XY}}{\sqrt{{se^{2}}_{XX}+{se^{2}}_{XY}\;-\;2\cdot r\cdot {se}_{XX}\cdot {se}_{XY}}}.$$

### Simulations

#### Data generating model

The data generating model is shown in Figure 1. Pseudo-men and women were simulated, drawing a date of birth at random from 1 January 1900 to 1 January 2020. Each pseudo-individual was followed over the course of 120 years or until their death, whichever came first. At birth, genotypes were assigned at three loci, each having a minor allele frequency of 10%. Two genotypes, Geno_StrokeMult_ and Geno_StrokeAdd_, incurred a risk on stroke only, and the other, Geno_Death_, incurred a risk on death via an unspecified pathway independent of stroke. The annual stroke risk was a function of sex, age, and genotypes given by

$$\begin{array}{l} Annual\;stroke\;risk\;\left(p_{stroke}\;|\;sex,age,Geno_{StrokeMult},Geno_{StrokeAdd}\right)\\ =\left(\frac{0.01}{1+\exp\left(-0.1\;*\;\left(age\;-\;60\right)\right)}\right)\;*\left(0.95*\;sex\right)\;*\;\left(\gamma_{effect}\;*\;Geno_{StrokeMult}\right)+\left(1\;-\;\gamma_{effect}\right)\;*0.003\;*\;Geno_{StrokeAdd}. \end{array}$$

The genetic effect of the genotype, $\gamma_{effect}$ , was simulated from 1.0 to 2.0 in increments of 0.1. An initial stroke event was drawn from a Bernoulli distribution with probability $\left(p_{stroke}\;|\;sex,age,Geno_{StrokeMult},Geno_{StrokeAdd}\right)$ , given that the subject had not died and had not previously experienced a stroke. If the binomial draw indicated a stroke at that age, an exact date of the stroke was randomly drawn from a uniform distribution of days in that year. The baseline annual risk of death was taken from the Social Security Administration Actuarial Life Tables (Lew, 1985). The mortality effect of Geno_Death_ was simulated using the same range of parameters, $\gamma_{effect}$ , and was a function of age. The relative increase in risk was assumed to be close to null at young ages and then increased over the lifetime until a pre-specified risk ratio. Stroke was assumed to increase the risk of death as a function of the time since the event, given by

$$Stroke\;Relative\;Risk\;\left(Stroke_{RR}\right)=1+\left(\frac{2}{\exp\left(YearsSinceStroke\right)/10}\right).$$

The resulting annual mortality risk was given by

$$\begin{array}{l} Annual\;Mortality\;Risk\;\left(P_{death}\;|\;sex,age,Geno_{Death},YearsSinceStroke\right)\\ =Base\;Risk_{sex,age}\;*\;\left(1+\frac{\left(\gamma_{effect}-\;1\;\right)}{1+\exp\left(-0.1\;*\;\left(age\;-\;60\right)\right)}\;*\;Geno_{Death}\right)\;*\;Stroke_{RR}. \end{array}$$

The given data generating model resulted in observations with seven features: date of birth, sex, $Geno_{StrokeMult}$ , $Geno_{StrokeAdd}$ , Geno_Death_, date of stroke, and date of death. Random draws of pseudo-individuals were prepared from the data generating model who were (1) alive as of 1 January 2020 and (2) over the age of 18 years on that date until 1,000 cases (defined as having a stroke prior to 1 January 2020) and 1,000 controls (defined as never having a stroke or having a stroke after 1 January 2020) were drawn. Each simulation scenario was replicated 1,000 times to obtain robust estimates of the mean of the estimated parameters and standard errors. The simulation study was performed using SAS (v9.4).

#### Genotypic models

Two genotypic models were simulated. The first modeled a constant relative risk over the lifespan, given by $\gamma_{effect}$ and parameterized as a risk ratio. The second modeled a constant additive risk over the lifespan given by a function of $\gamma_{effect}$ , as shown above. This model simulated a larger relative effect at younger ages than at older ages. It has been hypothesized that some genetic loci may have a disproportionate effect on stroke risk at younger ages *versus* older ages, and thus, genetic contributors to stroke risk may be easier to find (Kurki et al., 2023). For example, when $\gamma_{effect}=1.1$ , the early-onset locus had a relative risk of 1.6 at age 30 years, 1.1 at age 50 years, and 1.04 at age 70 years. This allows for a test of the ability of age-at-onset analyses to identify loci that have a larger relative effect early in life rather than later.

#### Target parameter

Given the above data generating model, it is trivial to determine the age at stroke for each pseudo-individual (date of stroke–date of birth). The target parameter was defined as the difference in the age of stroke between genotypes among cases.

$$Target\;Parameter\;\left(\theta \right)=E\left(\left(AAO\;|\;reference\;genotype\right)\right)\;-\;E\left(\left(\left.AAO\right|\;alternative\;genotype\right)\right).$$

Estimates of this target parameter were obtained using linear regression controlling for sex to approximate a common GWAS strategy. Genotypes were coded as 0, 1, and 2 to estimate the additive genetic model. Models were run for each of the simulated loci separately.

## Data Availability

The data presented in the study are deposited in the GWAS Catalog repository, accession numbers GCST90301368, GCST90301369, and GCST90301370.

## Key message statement

Utilizing a case-only GWAS design, we identified a significant association between the *APOE* locus, specifically the rs429358 variant, and AAO of ischemic stroke. This exonic variant in *APOE*, linked to the APOE-Є4 allele, exhibited a compelling correlation, with each copy of the allele associated with a 1.29-year earlier stroke onset. Notably, our findings, supported by a robust discovery cohort of 10,857 ischemic stroke cases and reinforced through meta-analysis with replication cohorts, shed light on a potential survival bias influencing the AAO. Although APOE-Є4 has previously been linked to increased mortality and stroke AAO, our simulation study suggests that this association may be independent of ischemic stroke itself, emphasizing the need for a nuanced interpretation in understanding the genetic factors influencing the age at onset in stroke cases.
